# 6,7-dimethoxy-1,2,3,4-tetrahydro-isoquinoline-3-carboxylic acid attenuates heptatocellular carcinoma in rats with NMR-based metabolic perturbations

**DOI:** 10.4155/fsoa-2017-0008

**Published:** 2017-05-12

**Authors:** Pranesh Kumar, Ashok K Singh, Vinit Raj, Amit Rai, Siddhartha Maity, Atul Rawat, Umesh Kumar, Dinesh Kumar, Anand Prakash, Anupam Guleria, Sudipta Saha

**Affiliations:** 1 Department of Pharmaceutical Sciences, Babasaheb Bhimrao Ambedkar University, Vidya Vihar, Raibareli Road, Lucknow 226025, India; 2 Department of Pharmaceutical Technology, Jadavpur University, Kolkata 700032; 3 Centre of Biomedical Research, SGPGIMS Campus, Raebareli Road, Lucknow 226014, Uttar Pradesh, India; 4 Department of Biotechnology, Babasaheb Bhimrao Ambedkar University, Vidya Vihar, Raibareli Road, Lucknow 226025, India

**Keywords:** 6,7-dimethoxy-1,2,3,4-tetrahydro-isoquinoline-3-carboxylic acid, diethylnitrosamine, hepatocellular carcinoma, NMR-based metabolomics

## Abstract

**Aim:**

6,7-dimethoxy-1,2,3,4-tetrahydro-isoquinoline-3-carboxylic acid (M1) was synthesized and evaluated for *in-vivo* antiproliferative action in diethylnitrosamine-induced hepatocarcinogenic rats.

**Materials & methods:**

The antiproliferative effect of M1 was assessed by various biochemical parameters, histopathology of liver and HPLC analysis. Proton nuclear magnetic resonance-based serum metabolic study was implemented on rat sera to explore the effects of M1 on hepatocellular carcinoma-induced metabolic alterations.

**Results:**

M1 showed protective action on liver and restored the arrangement of liver tissues in normal proportion. HPLC analysis displayed a good plasma drug concentration after its oral administration. Score plots of partial least squares discriminate analysis models exhibited that M1 therapy ameliorated hepatocellular carcinoma-induced metabolic alterations which signified its antiproliferative potential.

**Conclusion:**

M1 manifested notable antiproliferative profile, and warrants further investigation for future anticancer therapy.

## Lay abstract

Our previous study unveiled that an isoquinoline alkaloid (M1) isolated from *Mucuna pruriens* seeds had potent antiproliferative action on human hepatoma cells *in vitro*. In the present investigation we synthesized M1 and studied its *in vivo* antiproliferative effects in liver carcinogenic rats. To observe the effects of M1, various studies were conducted which include biochemical parameters of plasma and serum, histopathology of liver and HPLC analysis of plasma. In addition, ^1^H-NMR-based serum metabolic profiling was carried out to identify the effects of M1 on hepatocellular carcinoma-induced metabolic alterations. Our findings showed that compound M1 exemplifies remarkable efficacy against hepatocellular carcinoma.

Hepatocellular carcinoma (HCC) is considered the sixth most common malignancy and third most frequent leading cause of death [1]. It increases risk of death in developing countries, and 7.6 million people died from this cancer according to the WHO report in 2008 [2]. This is a most challenging situation for healthcare institutions as demand is increased for hepatic cancer treatment day to day with consequent economic repercussions. Drugs used for cancer chemotherapy are relatively toxic in nature and develop resistance during therapy. The molecular mechanisms of drug resistance may involve a variety of factors such as mutation of target genes and decreased drug concentrations in the cells due to renal toxicity [3–5]. Drugs from a natural origin are very few in number and synthetic drugs are costly and highly toxic to human body. Synthetic chemotherapeutic agents have been of proven efficacy for HCC treatment due to their chemoresistance [6]. Therefore, it is necessary to explore some newer anticancer drugs from plant sources to improve cancer prognosis and survival rate.

Natural products have been used for the treatment of hepatic cancer from ancient times but very few compounds isolated till date from natural origin are effective against hepatic cancer [7]. Our previous studies demonstrated that the ethyl acetate and methanol extracts of *Mucuna pruriens* (MP) seeds have potent antiproliferative action on human hepatoma cell line (Huh-7 cells) [8]. Recent investigation revealed that the seeds of MP are rich with isoquinoline alkaloids [9] and these alkaloids have a dramatic role in anticancer therapy [10,11]. Later, our group isolated one isoquinoline alkaloid from MP seeds (namely M1, 6,7-dimethoxy-1,2,3,4-tetrahydro-isoquinoline-3-carboxylic acid, Supplementary Figure 1) and demonstrated that M1 had dramatic role in antiproliferation against Huh-7 cells *in vitro* [12].

However, the *in vivo* antiproliferative action of M1 has not been reported thus far. Therefore, the present study was undertaken to find out the *in vivo* antiproliferative effect of M1 in hepatocarcinogenic albino Wistar rats. Various oxidative stress parameters, scanning electron microscopic (SEM) and histopathological studies of the liver tissues were performed to evaluate the protective effect of M1. ^1^H nuclear magnetic resonance (NMR)-based serum metabolic profiling was also performed to discover the metabolite modulation in HCC rats treated with M1.

## Materials & methods

### Drugs & reagents

Di-tert-butyl dicarbonate, sodium borohydride, *p*-toluene sulphonic acid and anhydrous methanol were procured from SD Fine Chemicals (Mumbai, India). Polyphosphoric acid and anhydrous methanol were purchased from Loba Chemicals (New Delhi, India). *p*-Iodoaniline, diethylnitrosamine (DEN) and 2,4-dinitrophenylhydrazine were procured from Sigma-Aldrich (Bengaluru, India). ALT and AST kit was acquired from the Transasia Bio-Medicals Pvt Ltd (Baddi, India). All other chemicals were obtained from Himedia (Mumbai, India). All the solvents and chemicals were of analytical grades with 99% purity and in house distilled water was used throughout the experiment.

### Procedure for synthesis of M1

#### Synthesis of 2-tert-butoxycarbonylamino-3-(3,4-dimethoxy-phenyl)-propionic acid (C1)

A solution of di-tert-butyl dicarbonate (3.4 ml, 15 mmol) in chloroform (10 ml) was added dropwise to a solution of 2-amino-3-(3,4-dimethoxyphenyl)-propionic acid (3.3 g, 15 mmol) in chloroform (10 ml) at 0°C. The mixture was stirred for another 24 h at room temperature and then it was concentrated to dryness. The residue was a colorless solid (Supplementary Figure 1; C1, 2.8 g, 84.90% yield).

#### Synthesis of 2-amino-3-(2-formyl-4,5-dimethoxy-phenyl)-propionic acid (C2)

C1 (2.6 g, 8 mmol), formic acid (1.2 ml, 30 mmol) and polyphosphoric acid (1.03 ml, 20 mmol) were taken in round bottom flask and heated at 60°C for 1 h. Later, the reaction mixture was kept at 4°C for overnight. A white colored precipitate was obtained (Supplementary Figure 1; C2, 1.67 g, 64.23% yield), filtered and dried.

#### Synthesis of 2-amino-3-(2-hydroxymethyl-4,5-dimethoxy-phenyl)-propionic acid (C3)

A mixture of C2 (1.4 g, 5.53mmol), sodium borohydride (0.41 g, 11.0 mmol) and anhydrous methanol (20 ml) was heated under nitrogen atmosphere for 2 h and then cooled at room temperature. The resulting crystal was filtered, washed with cold methanol (30 ml) and dried under vacuum at 40°C to get a white solid (Supplementary Figure 1; C3, 1.2 g, 85% yield).

#### Synthesis of 6,7-dimethoxy-1,2,3,4-tetrahydroisoquinoline-3-carboxylic acid (M1)

A mixture of C3 (1.1 g, 4.31 mmol) and *p*-toluene sulphonic acid (0.741 g, 4.31 mmol) in methanol were taken in 50 ml round bottom flask and stirred for 1 h at room temperature. Then, the reaction mixture was heated for 5 h and kept at 4°C for overnight. A white color precipitate was obtained which was recrystallized with methanol (Supplementary Figure 1; M1, 0.9 g, 81% yield).

### General experimental procedures for characterization of M1

UV spectrum was measured using UV-visible spectrophotometer (Cary-50 Bio, Varian Melbourne, Australia). Infrared spectrum (IR) was recorded in a FT-IR Spectrometer (Nicolet^™^ iS^™^50, MA, USA). Spectra from NMR spectroscopy were recorded using 800 MHz NMR spectrometer (Bruker, Rheinstetten, Germany) processed in Topspin-2.1. Direct-infusion mass spectroscopy data were acquired using a hybrid triple quadrupole linear ion trap mass spectroscopy equipped with an electrospray ionization source (2000 QTRAP, Applied Biosystems, CA, USA).

### Experimental animals

Male albino Wistar rats (80–120 g) were used for this experiment and Institutional Animal Ethical Committee approved the protocol previously (Approval No. SDCOP&VS/AH/CPCSE01/017/R2). Standard laboratory conditions (temperature 25 ± 5˚C and light/dark cycle of 12 h) were maintained with free access to commercial pellet diet and water *ad libitium.* Animals were kept for 1 week before experiment.

### Acute oral toxicity study

Acute oral toxicity study of M1 was performed as per revised Organization for Economic Cooperation and Development Guidelines 423. Animal Ethical Committee approved this study (Approval No. SDCOP&VS/AH/CPCSE01/017/R2). M1 was dissolved in 0.25% carboxymethyl cellulose (CMC) and administered orally at the dose of 25, 50, 100, 250 mg/kg body weight to albino Wistar rats for 4 weeks (n = 6) and the animals were observed every day for any toxic manifestations.

### Experimental design

All animals were randomly divided into five groups of six animals each. Drugs were suspended in 0.25% CMC, subjected to 28 days’ treatment orally and divided as follows: Group I: 0.25% CMC (2 ml/kg), Group II: DEN (8 mg/kg, intraperitoneally) [13], Group III: DEN+5-fluorouracil (5-FU, 10 mg/kg, intraperitoneally), Group IV: DEN+M1 (50 mg/kg, orally) and Group V: DEN+M1 (100 mg/kg, orally). The procedure to induce HCC was adopted from a paper by Song *et al.* [13] where it is shown that DEN produces HCC at 8 mg/kg intraperitoneal injection for 28 days to albino Wistar rats. After initial 1 week of adaptive inhabitation, all rats of group II–V were administered with DEN (8 mg/kg, intraperitoneally) for 28 days. After 28 days, 5-FU and M1 were given for 15 days as mentioned above for group III–V. At the end of the experimental period, animals were sacrificed by cervical decapitation and livers were dissected out immediately, rinsed in ice cold saline and stored at -20°C for further studies. The serum was collected, processed and stored for further analysis.

It should be noted that we have performed the *in vivo* experiments at 50 and 100 mg/kg doses as M1 is naturally isolated compound and usually 1/5th, and 1/10th of the safe dose is used for the *in vivo* studies (determined from acute toxicity studies using Organization for Economic Cooperation and Development Guidelines). Further, in most of the published papers on the naturally occurring compounds, the *in vivo* studies on albino Wistar rats were carried out at 50 and 100 mg/kg bodyweight doses [14,15].

### Estimation of plasma aspartate aminotransferase, alanine aminotransferase, biochemical estimations & bilirubin, biliverdin in liver

Liver function biomarkers like aspartate aminotransferase (AST), alanine aminotransferase (ALT) were also measured in serum using commercially available kit from Ebra Diagonesis Pvt Ltd, Germany [14]. The oxidative parameters like superoxide dismutase (SOD), thiobarbituric acid reactive substances (TBARS) [16], protein carbonyl (PC) [17], SOD [14], tissue catalase (CAT) [16] and glutathione (GSH) [18] levels were estimated in liver tissue in the similar experiment. The total protein content of each sample was measured using the Bradford reagent and bovine serum albumin was used as a standard. Conjugated bilirubin and biliverdin in liver were measured as per the following procedure stated earlier in the literature with slight modifications [19].

### Histopathological studies

Histopathological studies were also performed to find out the morphological changes of liver cells after M1 administration [20]. Liver tissues from each group were assessed for their morphological changes using hematoxylin and eosin staining. The tissues were preserved in 10% formalin overnight. Next day, the tissues again were superseded by 70% isopropanol overnight. Later, the tissues were exposed to isopropanol at various concentrations (70, 90 and 100%) and dehydrated by 100% xylene. The tissue samples were then embedded in bee’s wax and 5 μM sections were prepared by using microtome. Then, the tissues were succeeded by hematoxylin and eosin staining and observed under microscope (magnification 40X).

### SEM of liver samples

Liver tissue samples were collected (2–4 mm) and fixed in 2.5% glutaraldehyde for 2–6 h at 4°C for primary fixation. Then, the samples were washed with 0.1 M phosphate buffer for 15 min at 4°C. After that, 1% osmium tetroxide was used as a post fixation for 2 h at 4°C. Again, the samples were washed in 0.1 M phosphate buffer for 3 times at 15 min interval and kept at 4°C. Later, these samples were dehydrated with acetone at various concentrations (30, 50, 70, 90, 95 and 100%). After that, all specimens were air dried at room temperature and critical point drying (31.5°C at 1100 psi). Finally, samples were mounted on to the aluminium stubs with adhesive tape and observed for the morphological changes using scanning electron microscope (JEOL JSM-6490LV).

### ^1^H-NMR-based serum metabolic profiling

#### Sample preparation

All serum samples were thawed at room temperature, 250 μl of serum was taken and mixed with 250 μl of 0.9% saline sodium-phosphate buffer of strength 50 mM, pH 7.4 prepared in D_2_O. Then, the samples were centrifuged at 10,000 rpm for 5 min to remove any precipitates before acquiring the NMR data. A total 400 μl of the supernatant sample was used in 5 mm NMR tubes (Wilmad Glass, USA) for data acquisition with a co-axial insert containing the known concentration of TSP (sodium salt of 3-trimethylsilyl-(2,2,3,3-d4)-propionic acid) i.e. 0.1% was used as external standard reference to aid metabolite quantification for NMR experiment. Deuterium oxide (D_2_O; as a co-solvent and to provide a deuterium field/frequency lock) and sodium salt of trimethylsilylpropionic acid-d_4_ (TSP) used for NMR experiments, were purchased from Sigma-Aldrich (RI, USA).

#### NMR measurements

All NMR spectra were recorded at 298 K on Bruker Biospin Avance-III 800 MHz NMR spectrometer operating at proton frequency of 800.21 MHz, equipped with CryoProbe and an actively shielded gradient unit with a maximum gradient-strength output of 53 G/cm. The raw NMR data were processed in Topspin-2.1 (Bruker NMR data Processing Software). For each serum sample, one-dimensional ^1^H-NMR and diffusion edited ^1^H NMR spectra were recorded using the Carr–Purcell–Meiboom–Gill (CPMG) pulse sequence (cpmgpr1d, standard Bruker pulse program) with pre-saturation of the water peak through irradiating it continuously during the recycle delay (RD) of 5 sec, and the bipolar pulse pair longitudinal eddy current delay (BPP-LED) sequence, respectively. Each CPMG spectrum consisted of the accumulation of 128 scans and lasted for approximately 15 minutes. To remove broad signals from triglycerides, proteins, cholesterols and phospholipids, a total spin–spin relaxation time of 60 ms (n = 300 and 2τ=200δs) with a line broadening factor of 0.3 Hz was applied. For diffusion edited (DE)^1^H NMR pulse sequence, the square gradients of 70% of the maximum gradient strength (56 G/cm) and 2 ms duration (followed by a delay of 200 μs to allow for the decay of eddy currents) were used. Diffusion time of 120 ms was used to attenuate the signals from low molecular weight compounds without affecting the lipid signals. All the spectra were processed using Topspin-2.1 (Bruker NMR data Processing Software) using standard Fourier Transformation (FT) procedure following manual phase and baseline-correction. Prior to FT, each FID was zero-filled to 4096 data points and a sine-bell apodisation function was applied. After FT, the chemical shifts were referenced internally to methyl peak of L-lactate (at δ=1.33 ppm). All recorded spectra were visually inspected for acceptability and subjected to multivariate statistical analysis to identify the altered metabolic pattern.

#### Spectral assignment

For unambiguous assignment of various peaks in the^1^H CPMG NMR spectra, two-dimensional NMR (2D NMR) spectra were acquired for selected samples including ^1^H-^1^H total correlation spectroscopy (TOCSY) and ^1^H-^13^C heteronuclear single quantum correlation (HSQC). The chemical shifts were identified and assigned as far as possible, by comparing them with the chemical shifts available with the software Chenomx 8.1 (Chenomx Inc., Edmonton, Canada). The remaining peaks in the CPMG ^1^H NMR spectra were assigned using the existing databases and the literature reports such as The Human Metabolome Database (HMDB) [21–23].

#### Multivariate data analysis

NMR spectra were manually corrected for phase and baseline aberration using Topspin 2.1 (Bruker NMR data Processing Software). The CPMG (δ 0.5−8.5 ppm) and diffusion edited (δ 0.5−5.6 ppm) spectra were binned into 0.01 ppm integrated spectral buckets using AMIX package (Version 3.8.7, Bruker, Bio Spin). The region (δ 4.7–5.1) distorted due to water suppression were excluded from the CPMG and diffusion edited data to avoid the effects of imperfect water suppression. The data were obtained from AMIX after mean centering and normalization which was performed by dividing each data point by the sum of all data points present in the sample to compensate for the differences in concentration of metabolites among individual serum samples. The data were scaled using unit variance in which identical weight was given to all variables. The resulting data matrices were then exported into Microsoft Office Excel 2010 and used for multivariate analysis using open access web-based metabolomic data processing tool, named MetaboAnalyst [24] and Unscrambler X Software (Version 10.3, [CAMO, Oslo, Norway]). Principal component analysis was first performed on both the Carr-Purcell-Meiboom-Gill (CMPG) and diffusion edited (DE) datasets to identify the outliers. To further demonstrate the differences between the different groups, supervised partial least squares discriminate analysis (PLS-DA) was carried out to identify the metabolites significantly contributing to group differentiation. Model quality was assessed with R^2^ indicating the validity of models against overfitting and Q^2^ representing the predictive ability. Potential metabolites markers were identified from the loading plots (for PLS-DA) and the scores of variable importance on projections. The statistical significance of these metabolites was calculated by t-test (p < 0.05).

#### Determination of M1 in rat plasma

In a separate experiment, M1 was administered orally at 50 mg/kg bodyweight to albino Wistar rats (n = 3) and blood was collected from retro orbital plexus at 0.083, 0.25, 0.5, 1, 2, 4, 8, 12, 16, 24 and 36 h. After collection, blood was centrifuged at 10,000 rpm for 10 min, serum was separated and kept at -20°C for further HPLC determination [25,26].

#### Preparation of plasma standards & test samples

A 1 mg/ml stock solution of M1 was prepared in methanol. 2, 5, 10, 25, 50, 100, 200, 250 and 500 ng/ml working solutions were prepared from the stock solution in methanol. A 100 μl of blank plasma and 100 μl working solutions were taken in separate tubes and vortexed for 30 min. After that, the tubes were centrifuged at 13,000 rpm for 5 min and supernatant was taken into another tube and dried at 40°C overnight. Later, the tubes were reconstituted with 50 μl of methanol, vortexed for 10 min and 20 μl was injected for HPLC analysis. The final concentration of working solutions were 1, 2.5, 5, 12.5, 25, 50, 100, 125 and 250 ng/ml, respectively. Quality control (QC) samples were also prepared at concentrations of 3.0, 25.0 and 200.0 ng/ml, representing low, medium and high QC samples (LQC, MQC and HQC), respectively. Triplicate calibrants and QC samples were prepared at each concentration.

For test samples, 100 μl of test plasma and 100 μl methanol were taken in a test tube and vortexed for 30 min. The other procedure was similar to previously described method.

#### HPLC conditions

For the determination of M1 in plasma samples, chromatographic separations were performed in a Waters 2489 HPLC (MA, USA) equipped with Spherowsorb C18, 5μ, 4.6 × 250 mm column. The flow rate was at 1.0 ml/min and the mobile phases consisted of solvent A (formic acid containing water, pH 3.4) and solvent B (methanol). The elution condition was methanol:formic acid (70:30) and the total run time was 8 min. The HPLC was run through Empower software version 2 and absorbance was measured at 230 nm wave length. The detector used for estimation was Waters 2489 UV-Vis detector. The final data was calculated using Winnonlin version 1.5.3 software.

#### Statistical analysis

Statistical analysis was carried out using GraphPad Prism 5.0 (CA, USA). All results were expressed as mean ± standard deviation (SD). The data were analyzed by one-way analysis of variances followed by Bonferroni multiple comparison test. For biochemical estimations, statistical significance differences were considered with respect to toxic control (***p < 0.001; **p < 0.01).

## Results

### Characterization of M1

The characterization data of our synthesized M1 as well as corresponding naturally isolated M1 is shown in the Supplementary Material. On comparing both of these, the characterization data of our synthesized M1 were found to be in good agreement with the previously published data of naturally isolated M1.

### Acute oral toxicity study

No mortality and behavioral changes were observed up to 4 weeks. Various biological parameters such as ALT and AST in plasma, bilirubin and biliverdin in serum, and various biochemical parameters (SOD, CAT, PC, GSH, TBARS) in liver were measured. We found that there were no significant changes in any of these parameters following M1 treatment (dose ranging from 25 to 250 mg/kg) as compared with normal control (Supplementary Table 1). Results obtained from acute oral toxicity studies implied that M1 was safe up to 250 mg/kg body weight dose to albino Wistar rats. Therefore, we decided to perform HCC activity in 50 and 100 mg/kg bodyweight doses.

### Estimation of plasma AST, ALT, biochemical estimations & bilirubin, biliverdin in liver

Table 1 illustrates the activity of liver enzymes ALT and AST in all the experimental groups of rats. It is clear from Table 1 that both ALT and AST levels in plasma were increased significantly in toxic group (DEN-treated group-II) as compared with controls (group-I). The levels of these enzymes were normalized after the oral administration of M1 and both the ALT and AST values for the M1-treated groups were similar to that of the group-I (control) rats. The levels of ALT and AST were also found to be decreased in positive control group (5-FU-treated group), but the percentage decrease after M1 therapy (100 mg/kg dose) is more compared with 5-FU treatment.

**Table 1.** **Effect of M1 on alanine aminotransferase and aspartate aminotransferase in plasma after oral administration of 50 and 100 mg/kg for 15 days to diethylnitrosamine-induced albino Wistar rats.**

**Groups**	**ALT (U/L)**	**AST (U/L)**
Control	38.89 ± 3.53	26.52 ± 4.67
Toxic (DEN)	143.50 ± 2.70	112.56 ± 5.88
DEN+5-FU	68.36 ± 5.32***	61.58 ± 4.17***
M1 50 mg/kg	88.4 ± 3.18***	47.73 ± 1.76***
M1 100 mg/kg	58.04 ± 2.84***	40.95 ± 1.35***

Data represented as mean ± SD (n = 6). Statistically significant differences were observed between toxic control and test groups (one way-ANOVA followed by Bonferroni multiple comparison test [***p < 0.001]).

ALT: Alanine aminotransferase; AST: Aspartate aminotransferase; DEN: Diethylnitrosamine.

Table 2 shows the values for various oxidative stress parameters (SOD, CAT, GSH, TBARS and PC) in liver of all the groups. We observed that there was dramatic reduction of GSH in toxic control (∼4.60 μM) than normal control (∼8.07 μM). Improvement in GSH level was observed after M1 treatment (∼5.40 μM for 50 mg/kg and ∼6.69 μM for 100 mg/kg). Similar trend was observed for SOD, where we found that SOD level decreased to 20–30% in toxic control as compared with normal group. This level was again improved up to 70–80% in M1-treated groups. The CAT enzyme activity was found to be improved for both positive control and treated groups than toxic control (Table 2). Further the tissue malondialdehyde (MDA) and PC formation was also measured to evaluate the protective action of M1. The MDA formation was approximately 98 nM for toxic control which was reduced after M1 therapy (∼50 nM). Also, the PC formation was higher for toxic control (∼0.96 μM) which was reduced to approximately half for M1-treated rats (∼0.40 μM; Table 2). The positive control group (treated with 5-FU) also exhibited significant increase in SOD, CAT, GSH levels and significantly decreased in PC and MDA levels.

**Table 2.** **Effect of M1 on oxidative stress parameters in liver after oral administration of 50 and 100 mg/kg for 15 days to diethylnitrosamine-induced albino Wistar rats.**

**Groups**	**SOD (U/μg of protein)**	**CAT (nM of H_2_O_2_/min/μg of protein)**	**Reduced GSH (μM/μg of protein)**	**PC (μM/μg of protein)**	**MDA (nM/μg of protein)**
Control	8.34 ± 0.57	12.13 ± 0.86	8.07 ± 0.24	0.18 ± 0.01	23.15 ± 2.69
Toxic (DEN)	2.45 ± 0.39	4.85 ± 0.37	4.60 ± 0.27	0.96 ± 0.14	98.78 ± 4.45
DEN+5-FU	6.99 ± 0.43***	9.30 ± 0.35***	7.30 ± 0.39***	0.35 ± 0.07***	46.24 ± 0.26***
M1 50 mg/kg	5.92 ± 0.72***	6.51 ± 0.29***	5.40 ± 0.42**	0.46 ± 0.08***	51.60 ± 1.009***
M1 100 mg/kg	6.14 ± 0.17***	7.52 ± 0.7***	6.69 ± 0.30***	0.40 ± 0.02***	45.20 ± 1.05***

Data represented as mean ± SD (n = 6). Statistically significant differences were observed between toxic control and test groups (one way-ANOVA followed by Bonferroni multiple comparison test [***p < 0.001]).

CAT: Tissue catalase; DEN: Diethylnitrosamine; GSH: Glutathione; MDA: Malondialdehyde; PC: Protein carbonyl; SOD: Superoxide dismutase.

Both conjugated bilirubin and biliverdin levels in liver were increased in toxic groups (∼60 ng). As shown in Table 3, bilirubin level was decreased two times (∼35 ng) in both 5-FU and M1-treated rats. Similar trend was observed for biliverdin assay where we found that biliverdin concentration was decreased for M1-treated rats (∼16 ng) than toxic control (∼28 ng).

**Table 3.** **Effect of M1 on bilirubin and biliverdin in liver after oral administration of 50 and 100 mg/kg for 15 days to diethylnitrosamine-induced albino Wistar rats.**

**Groups**	**Bilirubin (ng/μg of protein)**	**Biliverdin (ng/μg of protein)**
Control	25.80 ± 2.70	15.11 ± 2.40
DEN	60.48 ± 3.55	28.62 ± 3.77
DEN + 5-FU	34.70 ± 2.12***	18.39 ± 3.11***
M1 50 mg/kg	50.59 ± 1.39***	21.19 ± 2.42**
M1 100 mg/kg	40.64 ± 3.47***	16.95 ± 1.96***

Data represented as mean ± SD (n = 6). Statistically significant differences were observed between toxic control and test groups (one way-ANOVA followed by Bonferroni multiple comparison test [***p < 0.001]).

DEN: Diethylnitrosamine.

### Histopathology and SEM analysis of liver

**Figure 1.**
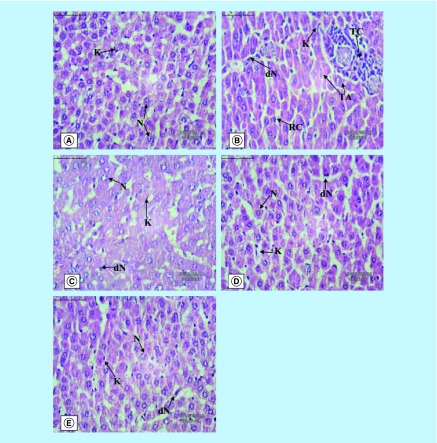
**The hepatic pathological changes in diethylnitrosamine-induced hepatocellular carcinoma rats (40X).** **(A)** Normal control, **(B)** toxic control (DEN), **(C)** positive control (DEN+5FU), **(D)** DEN+M1 (50 mg/kg), **(E)** DEN+M1 (100 mg/kg). We observed tumor cells, tumor anaplastic cells, dN and ruptured hepatic cell of Kupffer cell cell for DEN-treated rats. The normal tissue architecture was seen after oral administration of M1.

The histopathology of the liver tissue was examined under a light microscope and presented in Figure 1. Group I expressed (Figure 1A) the normal architecture of the liver cells with normal nucleus (N) and Kupffer’s cells (K). DEN Group-II (Figure 1B) depicted the degeneration of nucleus containing nucleolus (dN) and Kupffer’s cells (K) along with the ruptured hepatic cells (RC), tumor cells (TC) and tumor anaplastic cells (TA). Group III (Figure 1C) rats showed the normal histological appearance of liver cells (N & K). In Groups IV and V (Figure 1D & E), it was observed that the damaged liver architecture was altered, necrosis healed and the cellular degeneration was found to be lower depending upon the dose. SEM analysis expressed the similar trends where lesions were less prominent in M1-treated rats as compared with DEN group (Supplementary Figure 3).

### ^1^H-NMR method for serum metabolites profiling

Typical ^1^H CPMG NMR spectra of serum samples obtained from different groups are shown in Supplementary Figure 4. The NMR spectra showed signals mainly from lipids/lipoproteins (e.g., low-density lipoprotein [LDL], very low density lipoprotein [VLDL], unsaturated fatty acids, etc., and amino acids (e.g., alanine, valine, lysine, leucine, isoleucine, histidine, tyrosine, glutamine, glutamate and proline, among others). Other identified metabolites were glucose, choline, creatine, acetoacetate, acetone, acetate, citrate, lactate, *N*-acetyl and *O*-acetyl glycoproteins (NAG, OAG). We performed the multivariate data analysis to find out the HCC-induced metabolic alterations and further to reveal the effect of M1 treatment on these metabolic profile modulations.

### Metabolic changes in response to HCC in rats

**Figure 2.**
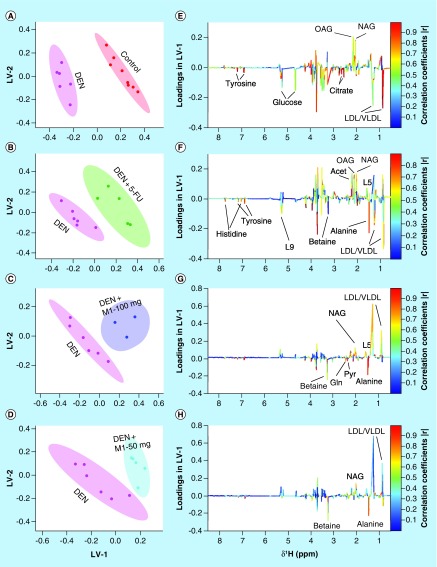
**Partial least squares discriminate analysis score plots.** The plots are derived from 1D CPMG ^1^H NMR spectra of rat serum samples between **(A)** control & DEN, **(B)** DEN & DEN+5-FU, **(C)** DEN & DEN+M1-100 mg and **(D)** DEN & DEN+M1-50 mg. **(E, F, G & H)** Shows the color coded coefficient loading plot corresponding to the PLS-DA analysis shown in **(A, B, C & D)**, respectively. The loading plots clearly demonstrate the metabolites responsible for the discrimination of the two groups in the corresponding score plots. Peaks in the positive direction (>0) indicate the metabolites which are more abundant in the groups in the positive direction of first principal component. Consequently, metabolites which are more abundant in the groups in the negative direction (<0) of first primary component are presented as peaks in the negative direction. DEN: Diethylnitrosamine; LDL: Low-density lipoprotein; NAG: *N*-acetyl glycoprotein; OAG: *O*-acetyl glycoprotein; PLS-DA: Partial least squares discriminate analysis; VLDL: Very LDL.

**Figure 3.**
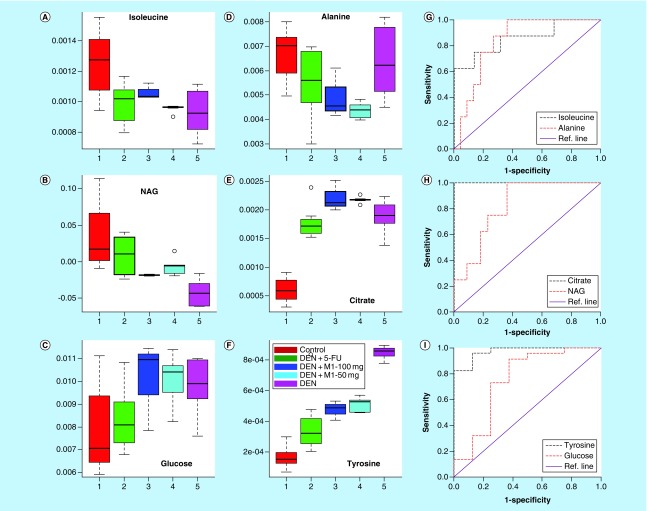
**Representative box-cum-whisker plots of the normalized integral areas of low molecular weight metabolites.** The plots are drawn from CPMG spectra **(A)** isoleucine, **(B)** NAG, **(C)** glucose, **(D)** alanine, **(E)** citrate and **(F)** tyrosine of control, DEN, DEN+5-FU, DEN+M1-100 mg and DEN+M1-50 mg rat sera. In the box plots, the boxes denote interquartile ranges, horizontal lines inside the box denote that the median, and bottom and top boundaries of boxes are 25th and 75th percentiles, respectively. Lower and upper whiskers are 5th and 95th percentiles, respectively. The corresponding ROC curves for these metabolites have also been displayed in **(G–I)**, respectively. DEN: Diethylnitrosamine; NAG: *N*-acetyl glycoprotein; ROC: Receiver’s operating characteristic.

**Figure 4.**
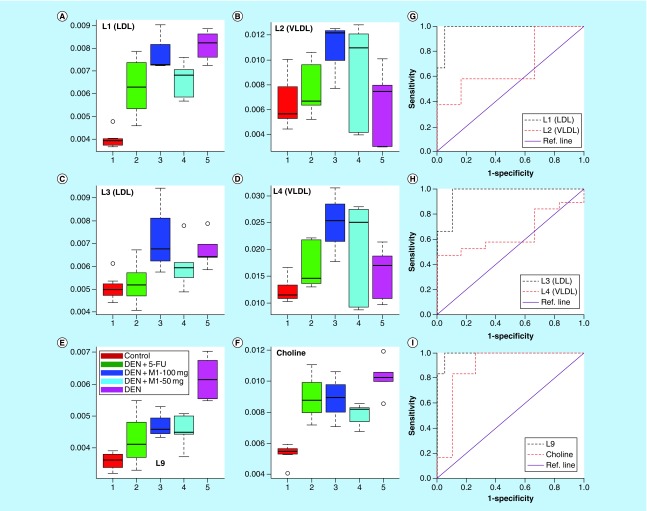
**Quantitative variations of low-density lipoprotein, very low-density lipoprotein, lipids and choline in control, diethylnitrosamine, DEN+5-FU, DEN+M1-100 mg and DEN+M1-50 mg rat sera represented as box-cum-whisker plots of normalized integral area.** Horizontal line inside the box is the median and bottom and top boundaries of boxes are 25th and 75th percentiles, respectively. Lower and upper whiskers are 5th and 95th percentiles, respectively. The corresponding ROC curves for these metabolites have also been displayed in **(G–I)**, respectively. DEN: Diethylnitrosamine; LDL: Low-density lipoprotein; ROC: Receiver’s operating characteristic; VLDL: Very LDL.

The principal component analysis score plot shown in Supplementary Figure 5 exhibited clear trend of clustering in different groups and no outlier sample was detected. To obtain satisfactory classification and select metabolite markers, pairwise PLS-DA analysis was further performed on NMR data matrices. The combined PLS-DA score plot for all groups (Supplementary Figure 6) and pairwise PLS-DA score plots (Figure 2A) showed that the clusters of DEN-treated rats are well separated from normal control group with a significantly higher quality of fit and predictability (R^2^ = 0.99, Q^2^ = 0.95; Table 4), indicating that significant metabolic changes were induced by DEN treatment. The corresponding loading plot in the first latent variable of PLS-DA is shown in Figure 2E which clearly revealed the metabolites responsible for discrimination of the two groups. The loading plots were color-coded according to the absolute value of correlation coefficients (|r|), where a hot-colored signal (red) indicated more significant contribution to class separation than a cold-colored one (blue). The key observed metabolic differences between the control and DEN group along with their chemical shifts, variable importance on projection score and p-value are listed in Table 5. Compared with control group, DEN-treated rats had significant elevation of lipids, lipoproteins (LDL), glucose, creatine, citrate, pyruvate, tyrosine and choline, together with decreased level for amino acids (such as leucine, isoleucine and glutamate), acetate, acetoacetate and NAG and OAG (Figures 3, 4 & Table 5).

**Table 4.** **Goodness-of-fit of the partial least squares discriminate analysis models obtained from 1D CPMG and diffusion-edited nuclear magnetic resonance-based analysis of rat serum samples.**

**Comparison**	**NMR spectra**	**R^2^X (cum)**	**R^2^Y (cum)**	**Q^2^ (cum)**	**Number of latent variables**
Control vs DEN	1D CPMG	0.79	0.99	0.95	3
DEN vs DEN+5-FU	1D CPMG	0.66	0.78	0.52	2
DEN vs DEN+M1-100 mg	1D CPMG	0.79	0.97	0.54	3
DEN vs DEN+M1-50 mg	1D CPMG	0.74	0.95	0.53	3
Control vs DEN	1D diffusion edited	0.66	0.96	0.90	2
DEN vs DEN+5-FU	1D diffusion edited	0.89	0.99	0.89	4
DEN vs DEN+M1-100 mg	1D diffusion edited	0.66	0.84	0.59	2
DEN vs DEN+M1-50 mg	1D diffusion edited	0.92	0.95	0.55	4

CPMG:Carr–Purcell–Meiboom–Gill; DEN: Diethylnitrosamine; NMR: Nuclear magnetic resonance; PLS-DA: Partial least squares discriminate analysis.

**Table 5.** **Key observed metabolic differences between the healthy control and diethylnitrosamine induced rat after M1 treatment.**

**Metabolites**	**Chemical shift**	**Variation in DEN with respect to control**	**VIP**	**p-value**	**Variation with respect to DEN**		
					**5-FU**	**M1-100mg**	**M1-50 mg**
Leu	0.96	↓	2.01	0.001	–	↑	↑
ILeu	1.00	↓	1.17	0.008	–	↑	–
Ala	1.48	–	–	–	↓	↓	↓
Lys	1.89	↑	1.86	0.002	↓	↓	↓
Ace	1.92	↓	1.79	0.04	↓	↓	↓
NAG	2.02	↓	3.76	0.003	↑	↑	↑
OAG	2.13	↓	3.46	0.03	↑	–	↑
Acet	2.23	–	–	–	↑	↑	↑
Acac	2.28	↓	1.56	<0.001	↑	↑	↑
Glu	2.35	↓	1.09	0.02	–	–	–
Pyr	2.37	↑	1.21	0.045	↓	↓	↓
Cit	2.52	↑	1.71	<0.001	–	↑	↑
Cr	3.03	↑	1.64	0.002	↑	↓	↑
Chol	3.22	↑	4.19	<0.001	↓	↓	↓
Betaine	3.265	–	–	–	↓	↓	↓
Glucose	3.23–3.92, 4.63, 5.21	↑	2.69–1.00	0.05	↓	–	–
Tyr	6.89	↑	2.05	<0.001	↓	↓	↓
L1 (LDL)	0.83–0.87	↑	5.6	<0.001	↓	↓	↓
L2 (VLDL)	0.87–0.90	–	–	–	↑	↑	↑
L3 (LDL)	1.23–1.27	↑	3.02	<0.001	↓	–	↓
L4 (VLDL)	1.27–1.30	–	–	–	↑	↑	↑
L6	2.00	↑	2.43	<0.001	↓	–	↓
L8	2.74	↑	2.05	<0.001	↓	–	–
L9	5.28	↑	2.65	0.001	↓	–	↓

Chemical shift, variation, VIP score and p-values of the individual biomarkers are given. p-values less than 0.05 were considered as significant. The metabolic differences between DEN and DEN+5-FU, DEN and DEN+M1-100 mg, and DEN and DEN+M1-50 mg have also been presented.

DEN: Diethylnitrosamine; LDL: Low-density lipoprotein; NAG: *N*-acetyl glycoprotein; OAG: O-acetyl glycoprotein; VLDL: Very low-density lipoprotein.

### Metabolic effects of M1 & 5-FU treatment

The combined PLS-DA score plot of all the five groups (Supplementary Figure 6) showed that 5-FU and M1 treatments shifted the state from DEN-treated group to back toward the control group. The pairwise score plots between M1, 5-FU-treated groups and DEN-treated group are shown in Figure 2B–D along with their loading plots (Figure 2F–H) which clearly revealed that the treatment groups are well separated from DEN-treated group. The significantly high values of R^2^ and Q^2^ (>0.5 as enlisted in Table 4) infer that all the PLS-DA models possessed a satisfactory fit with good predictive power. Further, we found that the metabolic alterations which were observed in DEN-treated group get ameliorated after the M1 treatment. For example, the metabolites which were increased in DEN-treated group such as lipids, LDL, glucose, pyruvate, choline, lysine and tyrosine get decreased in the M1-treated groups. Similarly, the levels of amino acids, NAG, OAG and acetoacetate were increased after M1 treatment. Similar amelioration of the metabolites was also observed after 5-FU treatment in HCC rats, that is, the metabolites which were increased in HCC rats get decreased after the 5-FU treatment and vice versa.

### Determination of plasma concentration of M1 using HPLC

**Figure 5.**
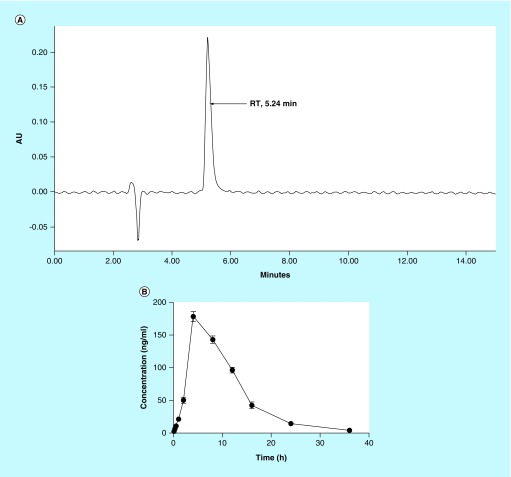
**High-performance liquid chromatography chromatogram and drug concentration profile in rat plasma.** **(A)** The retention time of M1. **(B)** Plasma drug concentration after single oral administration of M1 at 50 mg/kg dose at various time point (n = 3). RT: Retention time.

A linear regression performed over a range of 1–250 ng/ml yielded a correlation coefficient (r^2^) of >0.9 for M1 (Table 6). The accuracy of the assay was found to be within 81–92% and recovery of the samples was 67–83%. The retention time for M1 was 5.24 min (Figure 5A). As depicted in Table 6 and Figure 5B, the maximum plasma concentration (C_max_) and time required to reach the maximum concentration in plasma (T_max_) were 178.4 ng/mL and 4 h, respectively. The plasma concentration reached to 50% (t_1/2_) at 6.05 h. The total area under the curve after 36 h was 2065.05 ng.h/mL.

**Table 6.** **Various pharmacokinetic parameters after single oral administration of 50 mg/kg of M1 in rats (n = 3).**

**Parameters**	**Data**
Correlation coefficient (r^2^)	0.9246
Accuracy	81–92%
Recovery	67–83%
C_max_ (ng/ml)	178.4 ± 3.27
T_max_ (h)	4.0
T_1/2_ (h)	6.05
AUC_0-T_ (ng.h/ml)	2056.05 ± 26.22
AUC_0-α_ (ng.h/ml)	21688.88 ± 40.13
CL (min)	0.0005
MRT (min)	10.54

CL: Clearance; MRT: Mean retention time.

## Discussion

HCC is the third most frequent cause of cancer deaths in both developed and developing countries. The clinical outcome of HCC treatment remains unsatisfactory due to its resistance toward the presently usable chemotherapeutic agents. Furthermore, the usage of synthetic antiproliferative chemotherapeutic agents has declined due to their potential toxicity to human body. It is, therefore, necessary to investigate potential therapeutic agents for the clinical benefits among cancerous patients. Natural products are a good source from which to develop new medications for disease treatment as the compounds obtained from natural origin are often safe and less toxic in nature [3]. During the search for new HCC therapeutic agents, our group recently isolated M1 from MP seeds, which had good antiproliferative action on Huh-7 cells [12]. In this study, we investigated the *in vivo* antiproliferative action of M1 via its oral administration to hepatocarcinogenic albino Wistar rats. Results presented in the current study demonstrated the protective effect of M1 against DEN-induced HCC and are discussed in details further.

The levels of serum transaminases (AST and ALT) were found to be increased in DEN-treated rats as compared with the control group I indicating the hepatic damage in DEN group [16]. The enhanced levels of AST and ALT were lowered after the treatment significantly and dose-dependently with M1 inferring its ability to reduce hepatocarcinogenic features in HCC-bearing rats, similar to 5-FU. Various antioxidant parameters such as GSH, CAT and SOD were decreased significantly with simultaneous PC and MDA increased in DEN-treated rats, compared with that of control group. The alterations of these parameters reflect a good correlation of transformed cells in cancerous condition [27]. The levels of GSH, CAT and SOD were restored back to normalcy upon treatment with M1 in dose-dependent manner and in positive control group. Also the levels of MDA and PC were decreased significantly in M1 and 5-FU-treated groups, when compared with DEN rats. GSH is a tripeptide which is most abundant for all tissues including liver and plays major role in oxidation-reduction process to scavenge free radicals during oxidative damage [28]. M1-treated groups showed increase in GSH levels in dose-dependent manner and in 5-FU group bringing nearby to normal levels. Improvement in GSH levels after treatment revealed that M1 might be effective against DEN-induced liver damage. Further, the antioxidative enzymes SOD and CAT in liver provide protective defense against the reactive oxygen species. The enzyme CAT catalyzes the conversion of H_2_O_2_ to oxygen and water, thereby providing protection against reactive oxygen species and SOD neutralizes superoxide free radical in normal physiological situations [29]. These enzymes’ action is reduced due to the decreased expression of these antioxidants in DEN-induced HCC [30,31]. The restoration of the activities of SOD and CAT toward normalcy after the administration of M1 could be due to the antioxidant potency of M1 to scavenge reactive oxygen species.

Oxidation of lipids is an important parameter to measure oxidative stress-induced liver damage during cancerous condition [32]. The assessment of the degree of lipid peroxidation can be obtained by the measurement of MDA, which is one of the end products of lipid peroxidation. In the present study, oxidative stress leads to fourfold increase in the levels of MDA in DEN-treated rats. The decrease in the levels of MDA after treatment with M1 and 5-FU could be due to scavenging of the reactive free radicals involved in the peroxidation and, hence, may result in the inhibition of lipid peroxidation. Further, the carboxyl group of protein becomes oxidized due to formation of reactive oxygen species [33] and converted to PC which is also an important marker for oxidative stress-induced damage in cancerous cell. As depicted in the present study, PC levels were highly decreased in M1-treated rats, which again reveals the protective action M1 against DEN-induced hepatocellular damage. The upregulation of bilirubin and biliverdin, two unconjugated pigment metabolites, was also observed in DEN-treated rats, indicating the liver cells damage during HCC [34]. The levels of both the unconjugated pigment were restored to normal value after the treatment with M1 suggesting its protective action.

Further evidence of protective action was observed through histopathology and SEM analysis. Both analyses showed that the DEN-treated animals had irregular shaped nuclei with irregular cytoplasm, which might be due to excessive free radical generation during DEN administration [35]. M1 and 5-FU-treated groups demonstrated less ruptured and denatured cells (RC and DC) than toxic control, signifying their protective action against HCC. Similar trends were observed during SEM analysis.

NMR-based serum metabolomics coupled with multivariate statistical analysis was further carried out to investigate the HCC-induced metabolic alterations and further to evaluate the effect of M1 treatment on these alterations. A number of metabolites were identified that differed between DEN-treated group and normal control group. We found that HCC rat sera have higher levels of lipids, LDL lipoprotein, glucose, tyrosine, citrate, choline, pyruvate, creatine and lower levels of amino acids (such as leucine, isoleucine and glutamate), *N*- and *O*-acetyl glycoproteins, acetate and acetoacetate. The biological pathways involved in the metabolism of these metabolites and their biological roles were determined by enrichment analysis using MetaboAnalyst [36]. The metabolic pathways play a crucial role in the development of HCC that include the metabolism of glycine, serine and threonine, valine, leucine, isoleucine, pyruvate, phenylalanine, tyrosine, tryptophan, tyrosine and glyoxylate.

We observed significant increase in the levels of LDL, lipids and choline in HCC rats compared with normal rats. LDL mainly deliver cholesterol to cells, where it is used in membranes. Downregulated expression of LDL receptors in liver cells leads to elevation in levels of circulated LDL as it is not cleared from circulation due to the low functioning of LDL receptors [37]. Further fatty acids and lipids are also used for energy production such as β-oxidation, therefore, the increase in their level could be due to the results of energy requirement for cell membrane synthesis and fast-growing proliferation [38], and these alterations in lipogenesis might be an important factor for tumor development and growth. The decreased level of acetate, which is the end product of lipid metabolism, also reflects a disturbed lipid metabolism in HCC rats. Choline is an important intermediate of phospholipid metabolism which is an essential component in membrane structure and inflammatory mediators. High levels of choline could be due to the induced membrane transport of choline, upregulation of phospholipases and upregulation of choline kinase activity [39]. Choline is also known to attenuate immune inflammation through a cholinergic anti-inflammatory pathway; [40] therefore; the elevated choline level may be due to the activation of choline metabolism to dampen the inflammation associated with hepatic injury in HCC.

Significantly altered energy metabolism was observed in the DEN-treated rats. The citric acid levels was found to be elevated in the HCC rats suggesting an altered TCA tricarboxylic acid (TCA) cycle, which is in agreement with the commonly observed mitochondrial dysfunction in cancer [41]. The alterations in the citrate acid levels demonstrate the high energy demand as well as the altered enzyme activities in association with tumor growth. The decrease in the levels of acetoacetate in HCC rats also suggests the impairment in the TCA cycle and energy metabolism in liver mitochondria. The depleted levels of leucine and isoleucine are in concordance with the previous study on human HCC [42] which was reported to be associated with the reduced translation from succinyl-CoA because of the impaired TCA cycle as discussed above. Elevated levels of pyruvate were also found in the sera of HCC rats as compared with normal, which could be due to higher energy consumption or due to an increase in anaerobic cell respiration [42]. Serum creatine, which is a key intermediate in energy metabolism, was significantly elevated in HCC rats compared with normal rats and might be associated with increase in energy demand due to tumor growth. The levels of glucose was found to be higher in HCC rats compared with normal controls, whereas that of lactate, the end point product of the glycolysis pathway, was decreased (but not significant), suggesting dampened glycolysis in DEN-treated rats. Glutamine, lysine, and tyrosine, were also increased in the HCC rats which might be due to increased catabolism [43,44]. Taken together, due to aberrantly higher rates of cell proliferation, the rates of aerobic glycolysis, fatty acid synthesis and TCA cycle were higher in HCC rats to keep up with high energy and biomass demands as well as the altered enzyme activities in association with tumor growth.

The effect of M1 and 5-FU treatment on the metabolic alterations due to HCC as discussed above was investigated further. We found that the M1 treatment of DEN rats leads to the ameliorations of the HCC-induced metabolic alterations, signifying its antiproliferative properties. Similar ameliorations of the HCC-induced metabolic alterations were also observed in the standard 5-FU-treated group inferring that M1 treatment is equipotent to the standard 5-FU in restoration of altered metabolic patterns. Under the M1 treatment, metabolic markers (leucine, isoleucine, lysine, acetoacetate, pyruvate, creatine, choline, tyrosine, LDL lipoproteins and fatty acids) were modulated toward the level of normal controls. *N*-acetyl glycoproteins, an acute phase protein [45], have been found to be increased after M1 treatment in HCC rats. NAG protects the body from the oxidative stress because of anti-inflammatory and antioxidant properties. Thus, the increased levels NAG after M1 therapy might be related to suppress the inflammation and oxidative stress associated with hepatic injury in HCC rats.

Overall, the ameliorations of various metabolic markers after M1 treatment on HCC rats could be associated with the reconstruction of the cell membrane damages, improvement of the energy metabolism and repairing of the inflammation injury.

Further, the drug concentration in plasma was also measured to find out its absorption and tissue distribution. M1 had good C_max_ (178.4 ng/ml) which signified its good oral bioavailability and good plasma distribution. This result indicated that M1 had good absorption and, therefore, it showed its antiproliferative action on liver.

## Conclusion

While searching for new HCC agents, our previous study unveiled that the MP seeds had good antiproliferative action on Huh-7 cells [8] and this plant is rich with isoquinoline alkaloid [9]. In another of our studies, one isoquinoline alkaloid, namely M1, isolated from MP seeds, showed good antiproliferative action *in vitro* on Huh-7 cells through 3-(4,5-dimethylthiazol-2-y1)-2,5-diphenyl tetrazolium bromide (MTT) assay [12]. In the present study, *in vivo* antiproliferative effects of synthesized M1 in hepatocarcinogenic rats were investigated to evaluate whether the M1 therapy ameliorates the metabolic alterations caused by HCC and have protective action on liver tissue. The results suggested that M1 normalized various pathophysiological enzymes and increased antioxidant status, maintained normal tissue architecture and ameliorated metabolic profiling. The possible mechanisms could be related to reinstating the cell membrane damages, improving the energy metabolism and repairing the inflammation injury. HPLC analyses revealed that M1 had good plasma distribution after oral administration, signifying initiation of apoptosis in HCC. Altogether, this study provides the evidence that M1 might be effective against HCC.

## Future perspective

Despite intense research, the immense potential of natural products in cancer therapy still remains unexplored. Consequently, there is a desideratum to develop more potent and less toxic anticancer drugs from plant-derived products. In view of this, a plant-derived natural product (M1) was synthesized and tested for *in vivo* anticancer efficacy on its two oral doses – 50 mg/kg and 100 mg/kg. Altogether, the study showed that M1 might emerge as one of the potential leads for future drug design. The next step for this research is to perform the *in vivo* anticancer efficacy at its higher safe dose – 250 mg/kg – and observe the changes in various molecular biomarkers inside the cells during HCC condition and after M1 treatment. Last, this lead compound (M1) needs to go through its toxicity profiling for the better clarification of its suitability for the treatment of liver cancer.

**Summary points**

- Previously, an isoquinoline alkaloid (M1) isolated from *Mucuna pruriens* seeds was found effective against human hepatoma cell lines (Huh-7 cells) *in vitro*.
- In this experiment, M1 (6,7-dimethoxy-1,2,3,4-tetrahydro-isoquinoline-3-carboxylic acid) was synthesized and evaluated for *in-vivo* antiproliferative action in diethylnitrosamine-induced hepatocarcinogenic rats.
- Antioxidant study revealed the protective action of M1 on liver.
- Histopathology of liver displayed morphological changes and showed that M1 restored the arbitrary arrangement of liver tissues in normal proportion.
- Pharmacokinetic study through high-performance liquid chromatography analysis of plasma showed good oral bioavailability and plasma drug concentration after M1 administration.
- Nuclear magnetic resonance-based metabolic approach for hepatic carcinoma was employed to identify the alterations of metabolites/biomarkers during hepatic carcinoma and after M1 therapy. The study confirmed that M1 therapy ameliorated hepatocellular carcinoma-induced metabolic alterations which signified its antiproliferative potential.
