# Supplementary material for: 6,7-dimethoxy-1,2,3,4-tetrahydro-isoquinoline-3-carboxylic acid attenuates heptatocellular carcinoma in rats with NMR-based metabolic perturbations
Source: Future Sci OA. 2017 May 12;3(3):FSO202. doi: 10.4155/fsoa-2017-0008 (PMC5583658; doi:10.4155/fsoa-2017-0008)
Supplement: Supplementary file 1 [file fsoa-03-202-s1.docx]

**SUPPLEMENTARY MATERIALS**

**Ameliorative effects of 6,7-dimethoxy-1,2,3,4-tetrahydro-isoquinoline-3-carboxylic acid derived isoquinoline alkaloid in diethylnitrosamine induced hepatocellular carcinoma on antioxidant properties and metabolic alterations in rats**

Pranesh Kumar^a#^, Ashok K Singh^a#^, Vinit Raj^a^, Amit Rai^a^, Siddhartha Maity^b^, Atul Rawat^c,d^, Umesh Kumar^c^, Dinesh Kumar^c^, Anand Prakash^d^, Anupam Guleria^c^* and Sudipta Saha^a^*

*^a^Department of Pharmaceutical Sciences, Babasaheb Bhimrao Ambedkar University, Vidya Vihar, Raibareli Road, Lucknow 226025, India*

*^b^Department of Pharmaceutical Technology, Jadavpur University, Kolkata 700032*

*^c^Centre of Biomedical Research, SGPGIMS Campus, Raebareli Road, Lucknow 226014, Uttar Pradesh, India*

*^d^Department of Biotechnology, Babasaheb Bhimrao Ambedkar University, Vidya Vihar, Raibareli Road, Lucknow 226025, India*

^#^**Both authors contributed equally.**

**^*^Authors for Correspondence:**

**Dr. Anupam Guleria**

Assistant Professor

Centre of Biomedical Research (CBMR),

SGPGIMS Campus, Raebareli Road, Lucknow-226014

Uttar Pradesh, India

**Email:** [anuguleriaphy@gmail.com](mailto:anuguleriaphy@gmail.com)

**Dr. Sudipta Saha**

Department of Pharmaceutical Sciences,

Babasaheb Bhimrao Ambedkar University,

Vidya Vihar, Rai Bareli Road,

Lucknow-226025

Email: [sudiptapharm@gmail.com](mailto:sudiptapharm@gmail.com)

**Characterization of M1** (Synthesized product)

The structure of M1 was confirmed by FTIR, NMR and MS analyses (Supplementary material, Fig.S1 and S2). Melting point: 280-282°C. UV (MeOH) λ_max_ (log e): 210 (2.5), 230 (1.0) and 284 (0.5). FTIR (KBr) γ_max_: 3410 (-OH), 3021 (-CH=), 1617 (C=O), 1512 (C=C), 1387, 1215 CM^-1^. ^1^H NMR (800 MHz, DMSO-d_6_) (ppm): δ 3.04 (m, 1H, -CH_2_-), δ 3.27 (br m, 3H, -CH_2_-), δ 3.73 (br s, 6H, -OCH_3_), δ 4.27 (s, 1H, -NH-), δ 6.76 (s, 2H, -CH=). ^13^C NMR (200 MHz, DMSO-d_6_) (ppm): 27.80(C4), 43.90(C3), 54.14(C1), 55.13(-OCH3), 55.19(-OCH3), 109.25(C9), 111.51(C10), 119.21(C8), 122.39(C5), 148.74(C6), 149.22(C7), 169.63(-COOH). ESI-MS: 238.5.

**Characterization of M1** (Isolated product) [12]

Melting point: 281°C. UV (MeOH) λ_max_ (log e): 210 (2.5), 230 (1.0) and 284 (0.5). FTIR (KBr) γ_max_: 3410 (-OH), 3021 (-CH=), 1617 (C=O), 1512 (C=C), 1387, 1215 CM^-1^. ^1^H NMR (DMSO-d_6_) (ppm): δ 3.04 (m, 1H, -CH_2_-), 3.20 (m, 1H, -CH_2_-), 3.72 (s, 6H, -OCH_3_), 4.18 (m, 1H, -CH_2_-), 4.32 (m, 1H, -NH-), 6.84 (d, 2H, -CH=). ^13^C NMR (DMSO-d_6_) (ppm): 37.38 (C4), 53.08 (C3), 62.87 (C1), 65.25 (-OCH3), 65.27 (-OCH3), 119.25 (C9), 121.37 (C10), 129.67 (C8), 132.28 (C5), 157.53 (C6), 157.84 (C7), 179.66 (-COOH). ESI-MS: 238.5.

**Figure S1.** Synthesis of M1 (A = Di-tert-butyl dicarbonate, CHCl_3_, 0°C 1h, Room temperature 24 h; B = HCOOH, Polyphosphoric acid, 60°C, 1 h; C= NaBH_4,_ Anhydrous MeOH, 2 h; D= *p*-Toluene sulphonic acid, Anhydrous MeOH, 70°C, 5 h).

**C1**

**A**

**B**

**C2**

**C3**

**C**

**D**

**M1**

**Figure S2.** (A) ^13^C NMR, (B) ^1^H NMR and (C) MS of synthesized M1


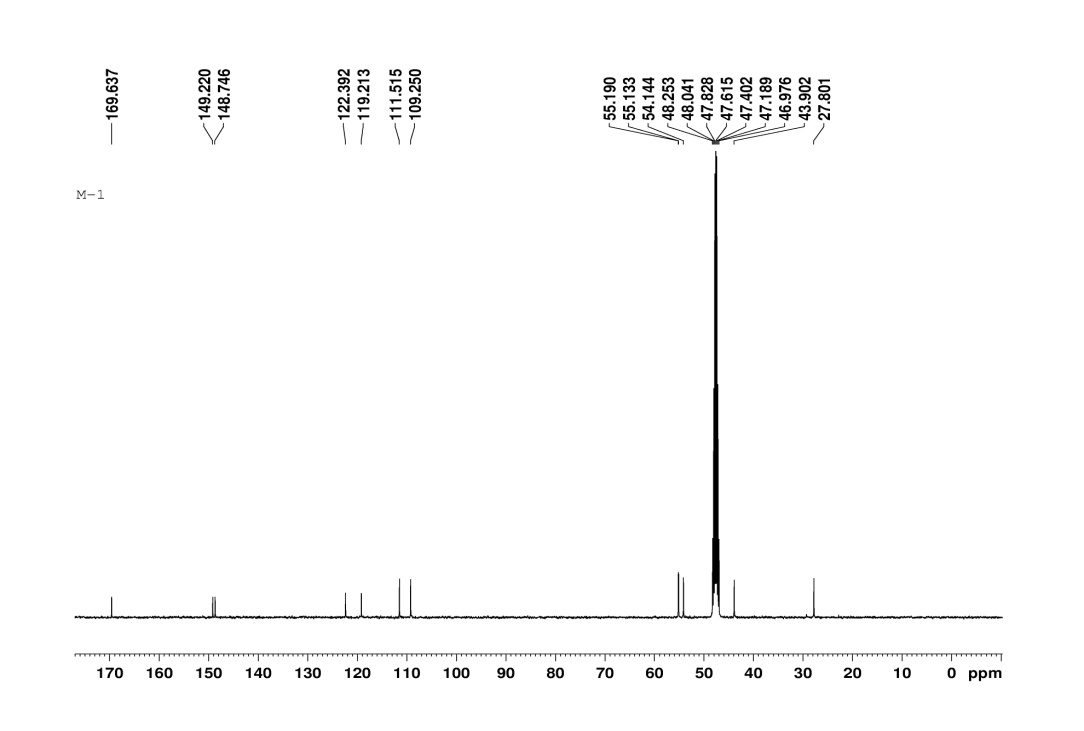


(A)


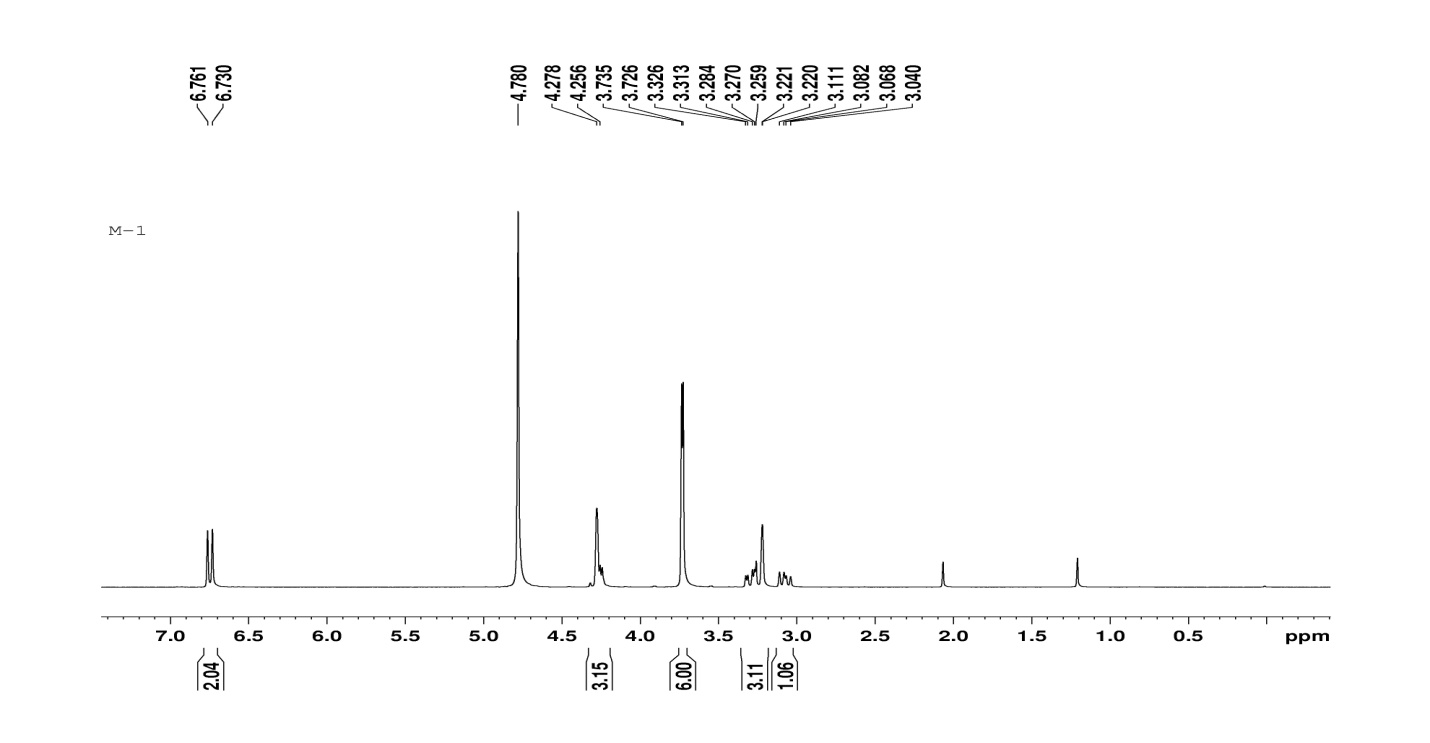


(B)


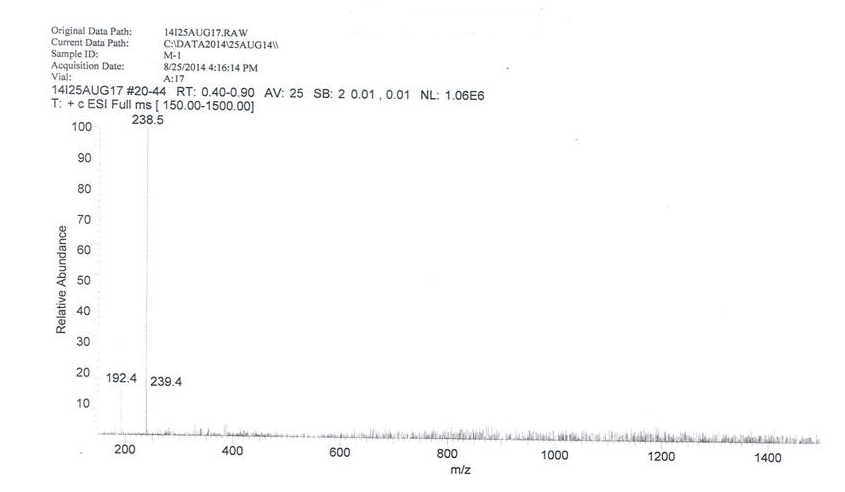


(C)

**Figure S3.** SEM photomicrographs of the liver tissues (X 2000). (A) Normal Control, (B) Toxic Control (DEN), (C) Positive Control (DEN+5FU), (D) DEN+ M1 (50 mg/kg), (E) DEN+M1 (100 mg/kg). Lesions were less prominent in M1 treated rats as compared to DEN groups.


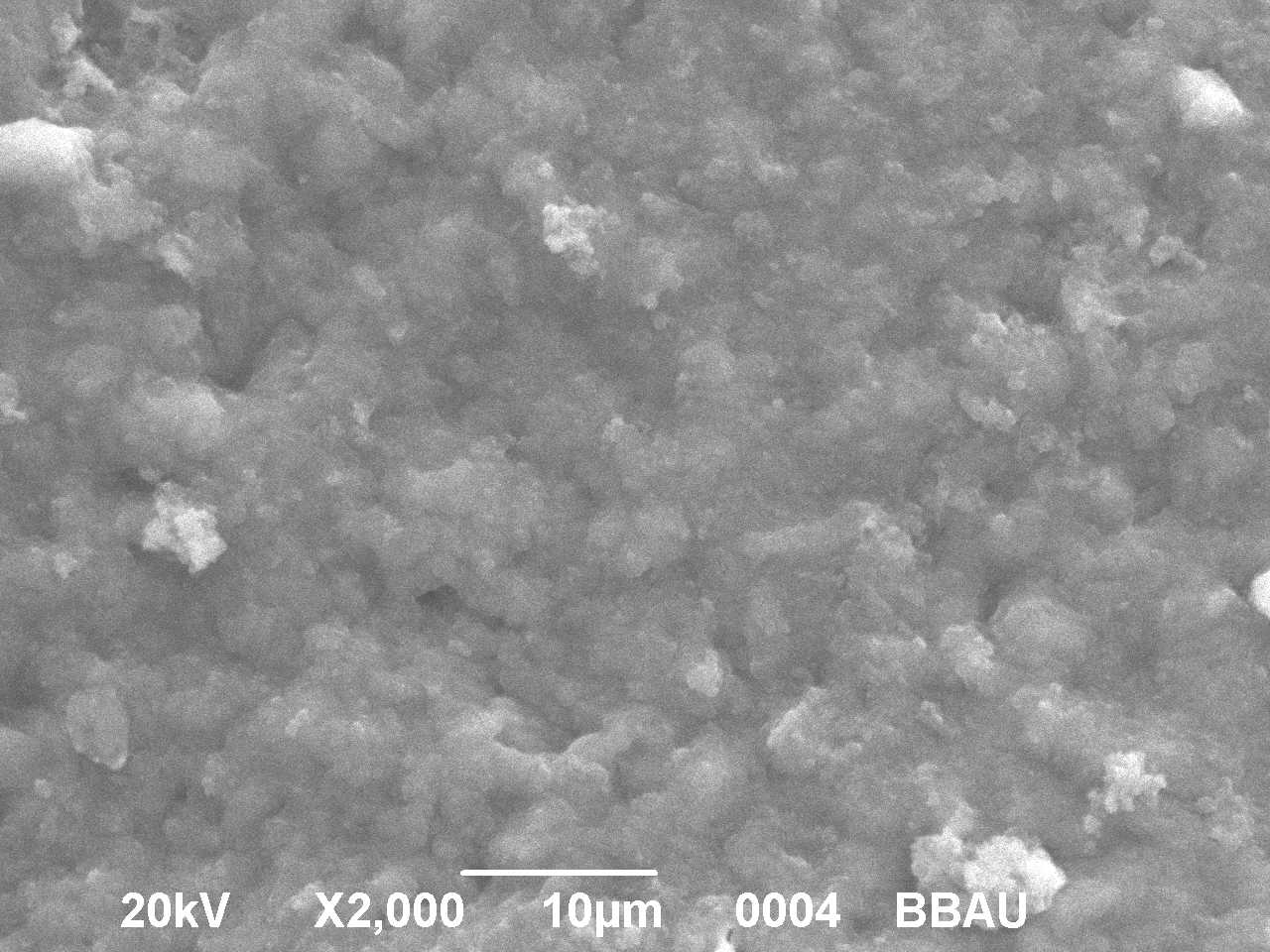

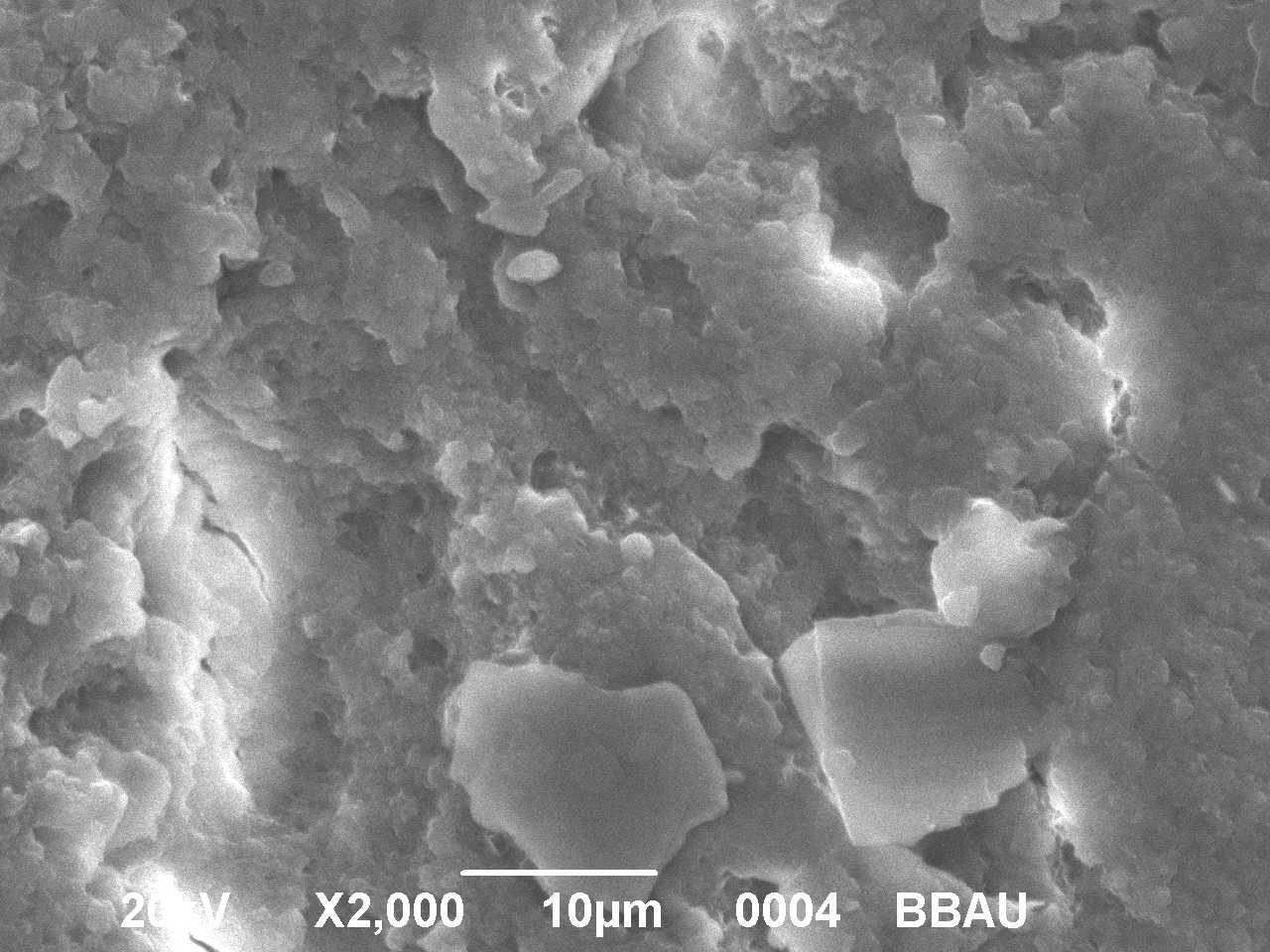


**A**

**B**


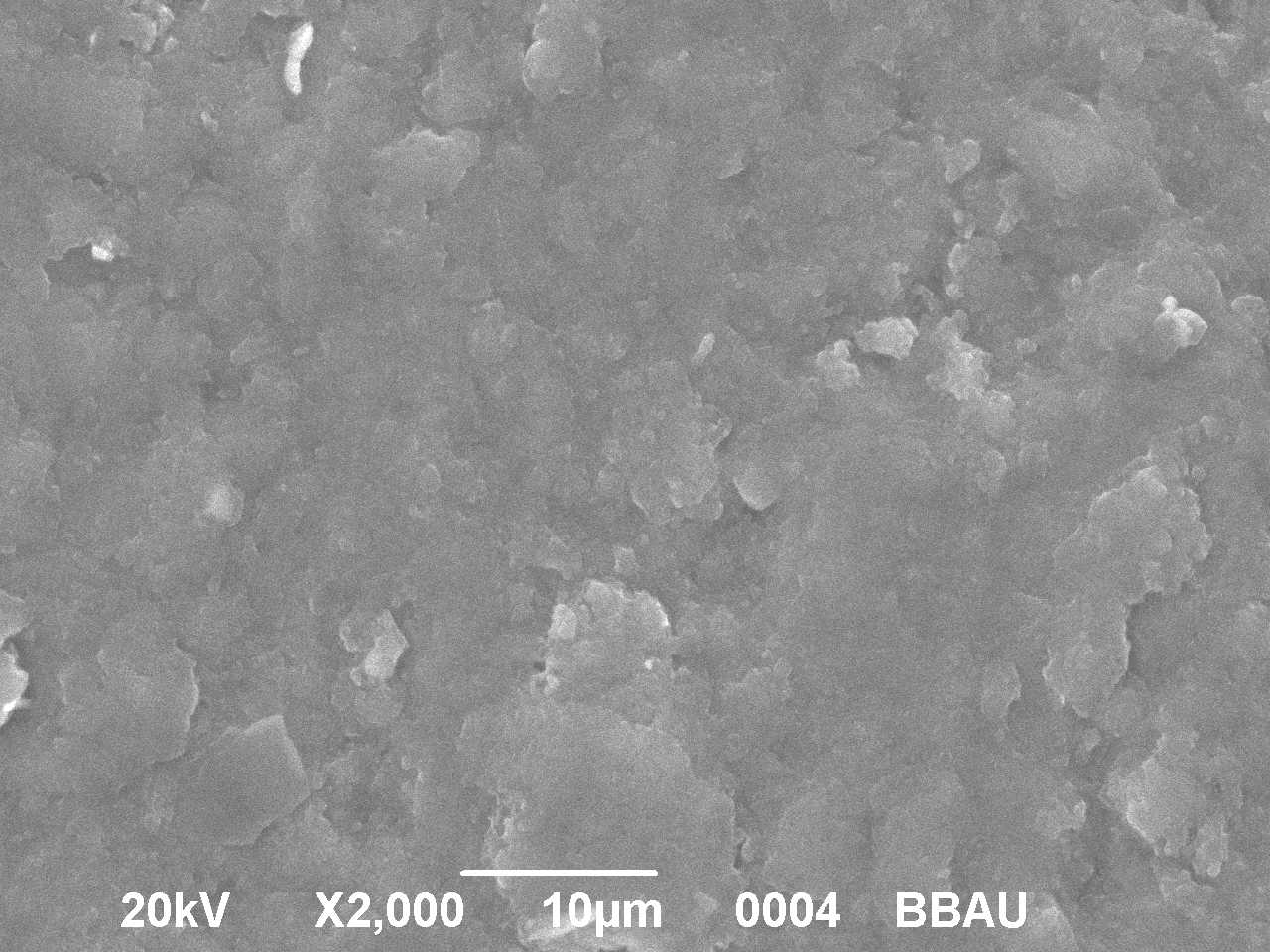

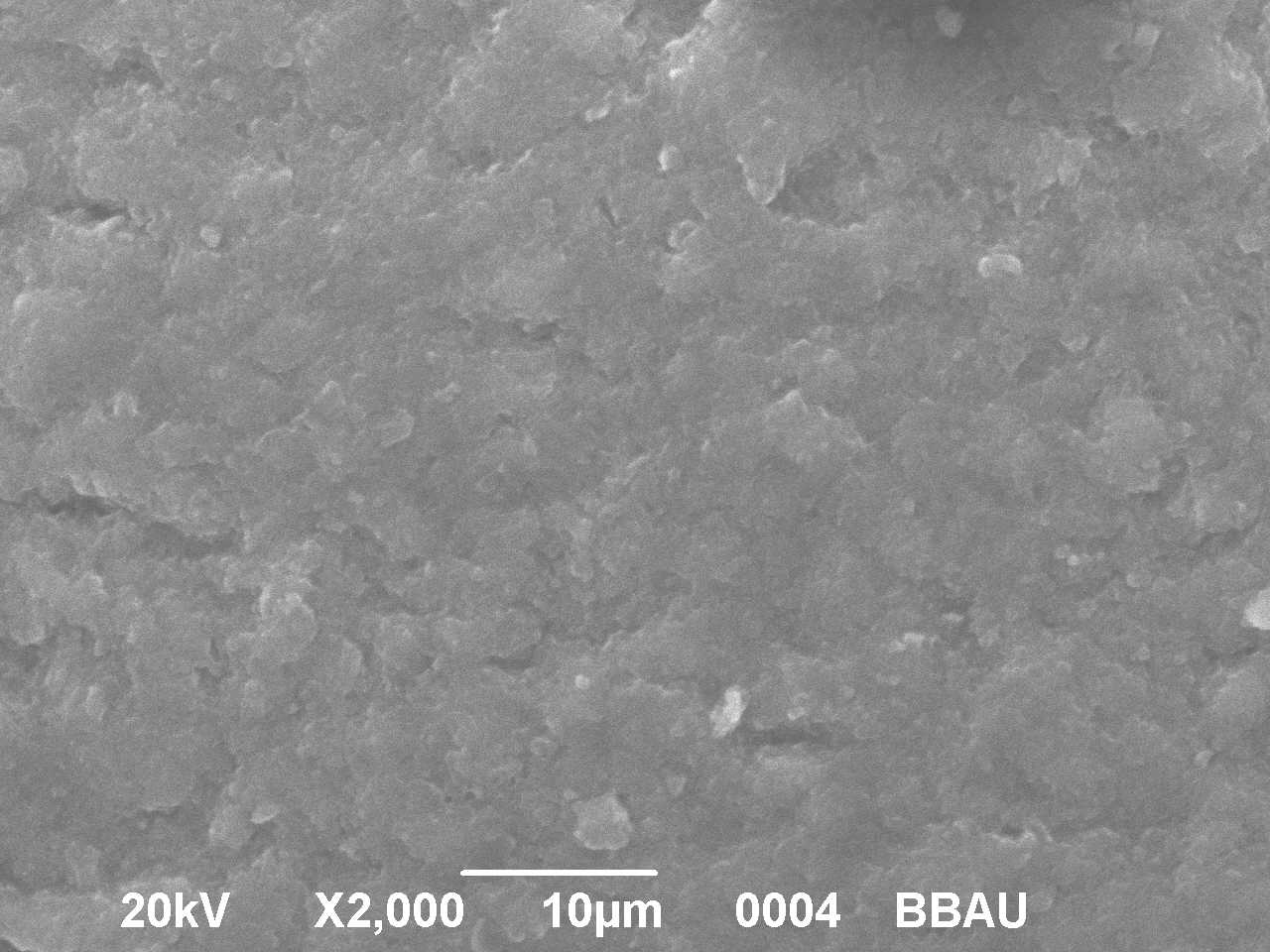


**D**

**C**


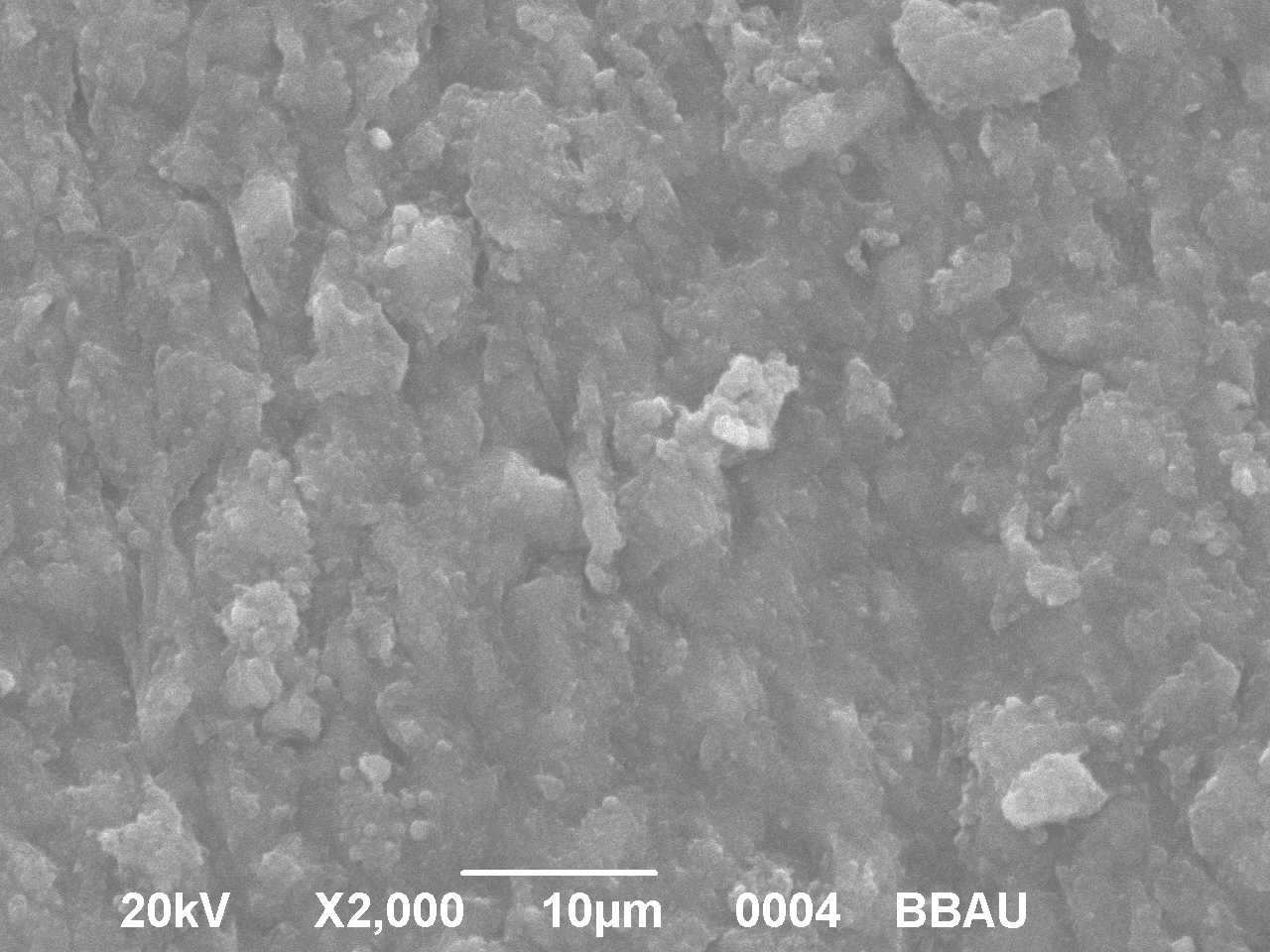


**E**

**Figure S4.** 800-MHz ^1^H CPMG NMR spectra (δ 0.5-4.7 and δ5.2-8.5) of rat serum obtained from (A) control, (B) DEN, (C) DEN+5-FU, (D) DEN+M1-100 mg, and (E) DEN+M1-50 mg. The region of δ 5.2-8.5 is magnified 8 times compared with the corresponding region of δ 0.5-4.7 for the purpose of clarity. **Key:** Ace: Acetate; Acac: Acetoacetate; Acet: Acetone; Ala: Alanine; Cit: Citrate; Chol: Choline; Cr: Creatine; For: Formate; Gln: Glutamine; His: Histidine; Ileu: Isoleucine; L: Lipid; L1/L2: CH_3_-(CH_2_)_n_- of LDL&VLDL; L3/L4: CH_3_-(CH_2_)_n_- of LDL&VLDL; L5: -CH_2_-CH_2_-C=O; L6: -CH_2_-CH=CH-; L7: -CH_2_-C=O; L8: =CH-CH_2_-CH=; L9: -CH=CH-; Lac: Lactate; Leu: Leucine; Lys: Lysine; NAG: N-acetyl glycoprotein; OAG: O-acetyl glycoprotein; Pyr: Pyruvate; Tyr: Tyrosine; Val: Valine; α-Glc: α-Glucose; β-Glc: β-Glucose.


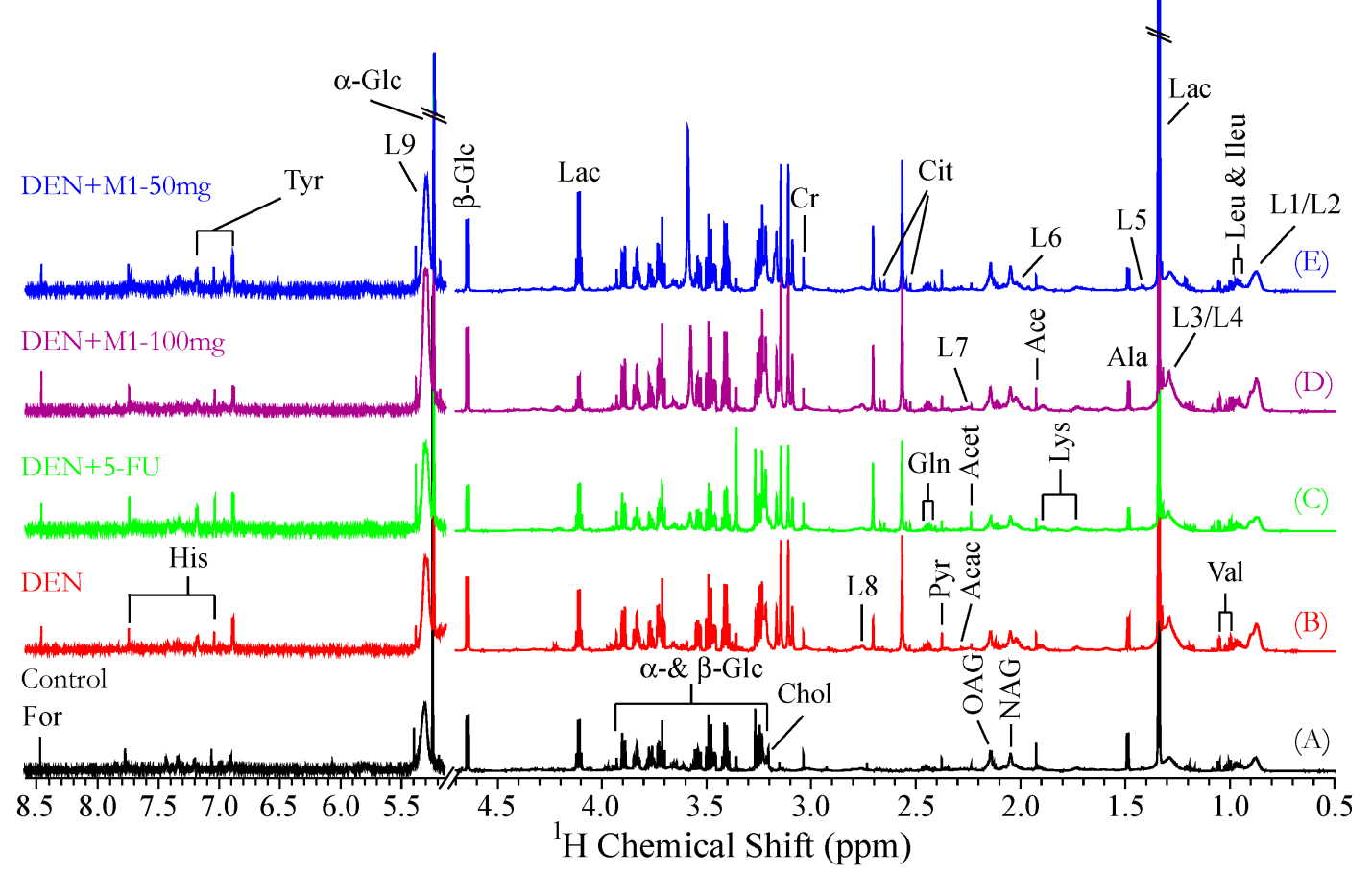


**Figure S5.** PCA score plot derived from 1D CPMG ^1^H NMR spectra of rat serum samples obtained from Control, DEN, DEN+5-FU, DEN+M1-100 mg and DEN+M1-50 mg groups.

**
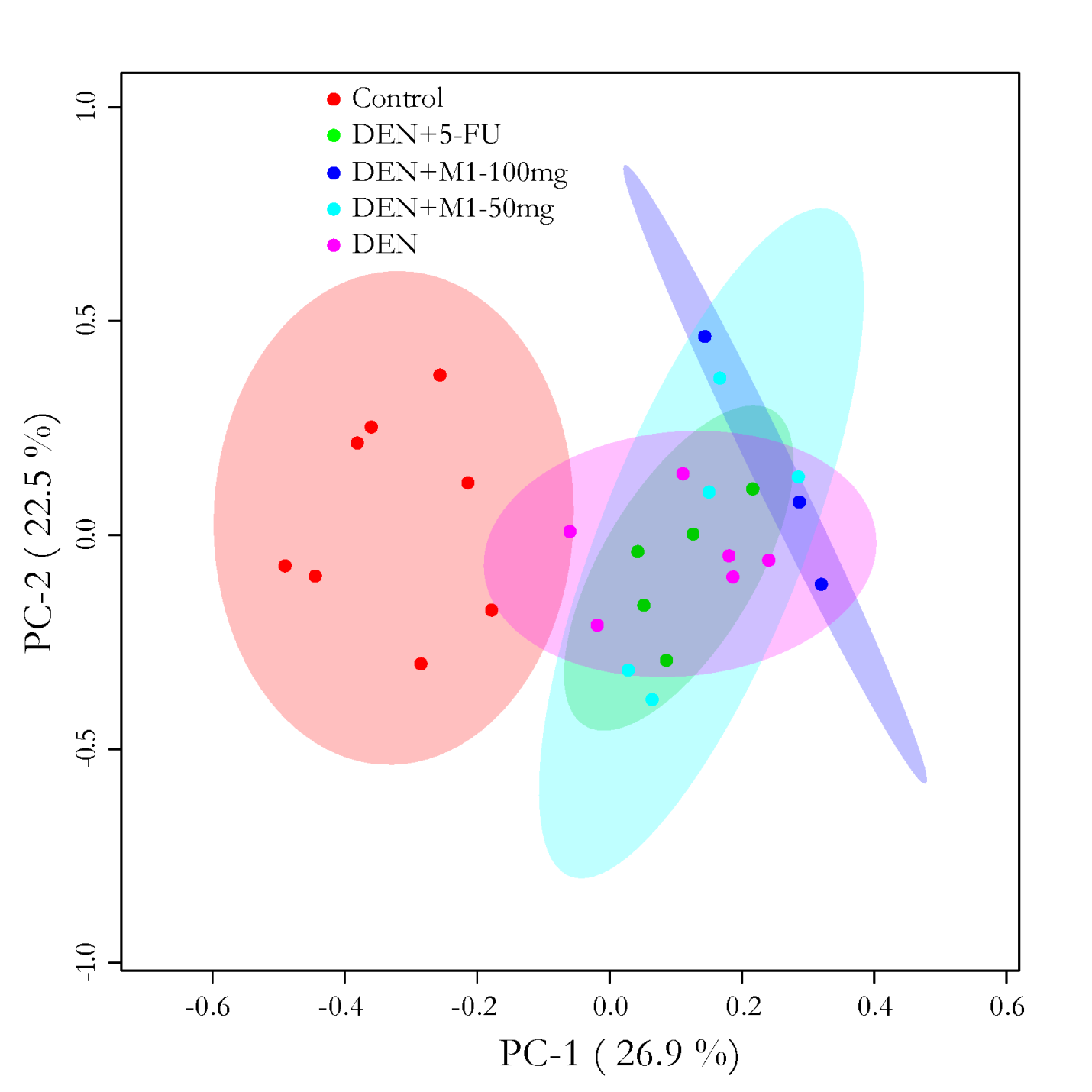
**

**Figure S6.** PLS-DA score plot derived from 1D CPMG ^1^H NMR spectra of rat serum samples obtained from Control, DEN, DEN+5-FU, DEN+M1-100 mg and DEN+M1-50 mg groups.


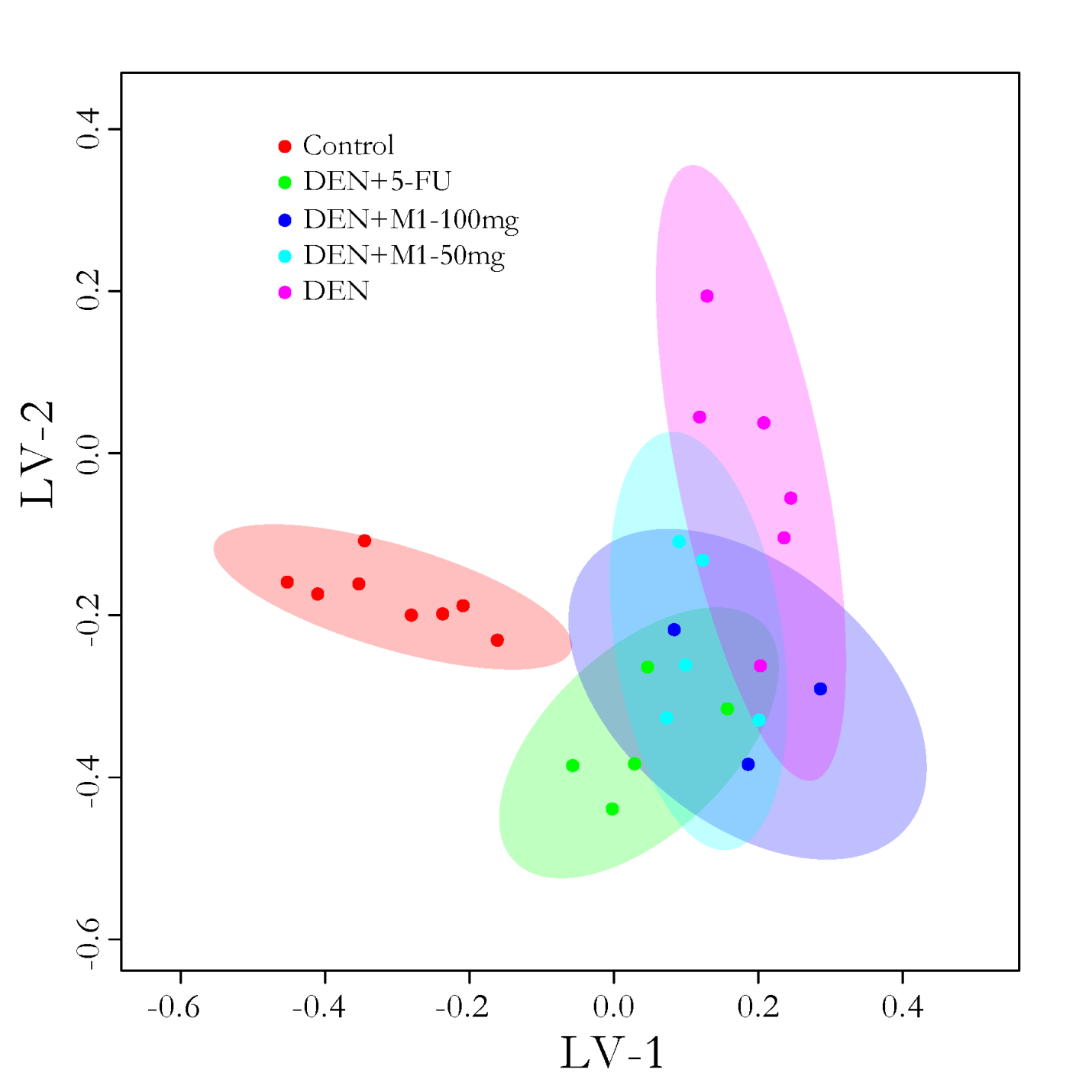


**Table S1.** Effect of M1 on (A) ALT and AST in plasma (B) oxidative stress parameters in liver and (C) bilirubin and biliverdin in liver after oral administration of 25, 50, 100 and 250 mg/kg for 15 days to albino Wistar rats.

(A)

| **Groups** | **ALT (U/L)** | **AST (U/L)** |
| --- | --- | --- |
| **Control** | 31.25 ± 4.11 | 15.23 ± 2.15 |
| **M1 (25 mg/kg)** | 35.70± 1. 77* | 11.33 ± 3.67* |
| **M1 (50 mg/kg)** | 33.16± 4.22 | 11.90 ± 1.69* |
| **M1 (100 mg/kg)** | 33.24 ± 1.57 | 14.33 ± 1.28 |
| **M1 (250 mg/kg)** | 35.11 ± 2.84 | 14.92 ± 3.52 |

(B)

| **GROUPS** | **SOD (U/μg of protein)** | **CAT(nM of H_2_O_2_ /min/μg of protein)** | **Reduced GSH (µM/μg of protein)** | **PC (µM/μg of protein)** | **MDA (nM/μg of protein)** |
| --- | --- | --- | --- | --- | --- |
| **Control** | 11.32 ± 1.98 | 7.98 ± 1.01 | 4.38 ± 1.09 | 0.54 ± 0.07 | 12.67 ± 1.18 |
| **M1 (25 mg/kg)** | 14.27 ± 2.88 | 8.22 ± 1.91 | 4.94 ± 1.17 | 0.68 ± 0.17* | 9.25 ± 2.87* |
| **M1 (50 mg/kg)** | 9.78 ± 1.17 | 6.94 ± 2.06 | 5.42 ± 1.34 | 0.62 ± 0.05 | 10.11 ± 0.96** |
| **M1 (100 mg/kg)** | 8.97 ± 1.95 | 8.45 ± 1.59 | 5.78 ± 1.65 | 0.49 ± 0.07 | 11.22 ± 1.91 |
| **M1 (250 mg/kg)** | 9.12 ± 1.65 | 7.38 ± 1.28 | 6.22 ± 1.07* | 0.48 ± 0.08 | 12.33 ± 2.17 |

(C)

| **Groups** | **Bilirubin (ng/µg of protein)** | **Bilverdin (ng/µg of protein)** |
| --- | --- | --- |
| **Control** | 17.90±2.55 | 6.14± 1.23 |
| **M1 (25 mg/kg)** | 19.24±1.27 | 7.15±0.78 |
| **M1 (50 mg/kg)** | 19.11±3.19 | 5.99±1.04 |
| **M1 (100 mg/kg)** | 18.24±2.55 | 7.62±1.45 |
| **M1 (250 mg/kg)** | 20.13±2.56 | 7.91±0.99* |

Data represented as mean±SD (n=6). Statistically significant differences were observed between control and test groups [Paired t-Test, *p<0.05, **p<0.01].
